# Early economic evaluation of MRI-guided laser interstitial thermal therapy (MRgLITT) and epilepsy surgery for mesial temporal lobe epilepsy

**DOI:** 10.1371/journal.pone.0224571

**Published:** 2019-11-20

**Authors:** Elysa Widjaja, Tina Papastavros, Beate Sander, Carter Snead, Petros Pechlivanoglou

**Affiliations:** 1 Institute of Health Policy, Management and Evaluation, University of Toronto, Toronto, Canada; 2 Diagnostic Imaging, Hospital for Sick Children, Toronto, Canada; 3 Division of Neurology, Hospital for Sick Children, Toronto, Canada; 4 Leslie Dan Faculty of Pharmacy, University of Toronto, Toronto, Canada; 5 Toronto Health Economics Technology Assessment, University Health Network, Toronto, Canada; 6 Institute of Clinical Evaluative Sciences (ICES), Toronto, Canada; 7 Public Health Ontario, Toronto, Canada; 8 Child Health Evaluative Sciences, Hospital for Sick Children, Toronto, Canada; Icahn School of Medicine at Mount Sinai, UNITED STATES

## Abstract

**Background:**

MRI-guided laser interstitial thermal therapy (MRgLITT) is a new minimally invasive treatment for temporal lobe epilepsy (TLE), with limited effectiveness data. It is unknown if the cost savings associated with shorter hospitalization could offset the high equipment cost of MRgLITT. We examined the cost-utility of MRgLITT versus surgery for TLE from healthcare payer perspective, and the value of additional research to inform policy decision on MRgLITT.

**Methods:**

We developed a microsimulation model to evaluate quality adjusted life years (QALYs), costs, and incremental cost-effectiveness ratio (ICER) of MRgLITT versus surgery in TLE, assuming life-time horizon and 1.5% discount rate. Model inputs were derived from the literature. We conducted threshold and sensitivity analyses to examine parameter uncertainties, and expected value of partial perfect information analyses to evaluate the expected monetary benefit of eliminating uncertainty on probabilities associated with MRgLITT.

**Results:**

MRgLITT yielded 0.08 more QALYs and cost $7,821 higher than surgery, with ICER of $94,350/QALY. Influential parameters that could change model outcomes include probabilities of becoming seizure-free from disabling seizures state and returning to disabling seizures from seizure-free state 5 years after surgery and MRgLITT, cost of MRgLITT disposable equipment, and utilities of disabling seizures and seizure-free states of surgery and MRgLITT. The cost-effectiveness acceptability curve showed surgery was preferred in more than 50% of iterations. The expected monetary benefit of eliminating uncertainty for probabilities associated with MRgLITT was higher than for utilities associated with MRgLITT.

**Conclusions:**

MRgLITT resulted in more QALYs gained and higher costs compared to surgery in the base-case. The model was sensitive to variations in the cost of MRgLITT disposable equipment. There is value in conducting more research to reduce uncertainty on the probabilities and utilities of MRgLITT, but priority should be given to research focusing on improving the precision of estimates on effectiveness of MRgLITT.

## Introduction

Approximately one third of patients with epilepsy remain resistant to pharmacotherapy despite treatment with two or more antiepileptic drugs (AEDs) and are known to have drug resistant epilepsy [1]. Temporal lobe epilepsy (TLE) is the most common type of drug resistant epilepsy in adults. Patients with drug resistant TLE generally experience greater morbidity, have poorer quality of life, access more healthcare resources and are at elevated risk of sudden unexplained death relative to those with well-controlled epilepsy [2]. Resective epilepsy surgery is a potentially curative intervention, and could render these patients seizure-free. Two randomized controlled trials have established the superiority of epilepsy surgery over medical management for patients with drug resistant TLE, not only in controlling seizures, but also in improving quality of life [3, 4]. The American Academy of Neurology recommends epilepsy surgery in patients with focal drug resistant epilepsy who do not respond to AEDs [5]. Despite mounting evidence supporting the effectiveness of epilepsy surgery, patients with suspected focal drug resistant epilepsy are frequently not referred for epilepsy surgery evaluation [6]. Fear or misconceptions of the risk of epilepsy surgery due to its invasive nature may contribute to underutilization of this treatment [7].

Advances in magnetic resonance imaging (MRI) with real-time temperature monitoring, along with technical improvements in laser ablation, have allowed precise ablation through a small burr-hole in the skull, leading to the development of a minimally invasive epilepsy treatment, MRI-guided laser interstitial thermal therapy (MRgLITT). This novel treatment has been proposed as an alternative to epilepsy surgery in addressing some of the concerns and misconceptions about the risk associated with surgery. Existing literature suggests that there are advantages of MRgLITT, including faster recovery and shorter hospital stay compared to epilepsy surgery [8–10]. However, it is not known if the cost saving associated with reduced hospitalization could offset the high capital investment and disposable equipment costs associated with MRgLITT. Furthermore, MRgLITT is a relatively new intervention, with a paucity of effectiveness evidence on MRgLITT versus epilepsy surgery. The aims of this study were to conduct an early economic evaluation of MRgLITT relative to epilepsy surgery in adults with drug resistant TLE from a healthcare payer perspective, and to determine the value of acquiring additional research evidence to inform clinical and policy decision on MRgLITT versus epilepsy surgery, also known as the value of information (VOI).

## Methods

Research ethics board approval was not required for this decision modeling.

### Decision model

We conducted a model-based cost-utility analysis using a probabilistic microsimulation model, comparing MRgLITT relative to epilepsy surgery in adults with drug resistant TLE who have undergone the same pre-surgical diagnostic evaluation, and were deemed eligible for MRgLITT or epilepsy surgery. We followed a hypothetical cohort of adults with an average age of 35.8 years (standard deviation of 1.2 years), based on the age distribution from a population-based study of adults undergoing epilepsy surgery [11]. All analyses were done from the Canadian healthcare payer perspective, which included all direct healthcare costs. The outcomes of interest were quality adjusted life years (QALYs), incremental QALYs, healthcare costs, incremental costs, and incremental cost-effectiveness ratio (ICER). ICER is the ratio of the difference in mean costs between the two treatment strategies to the difference in mean effectiveness. A life-time time horizon and yearly cycle length were used. A discount rate of 1.5% was applied to both costs and health effects, in accordance with the Canadian Agency for Drugs and Technologies in Health (CADTH) guideline [12].

**Fig 1:**
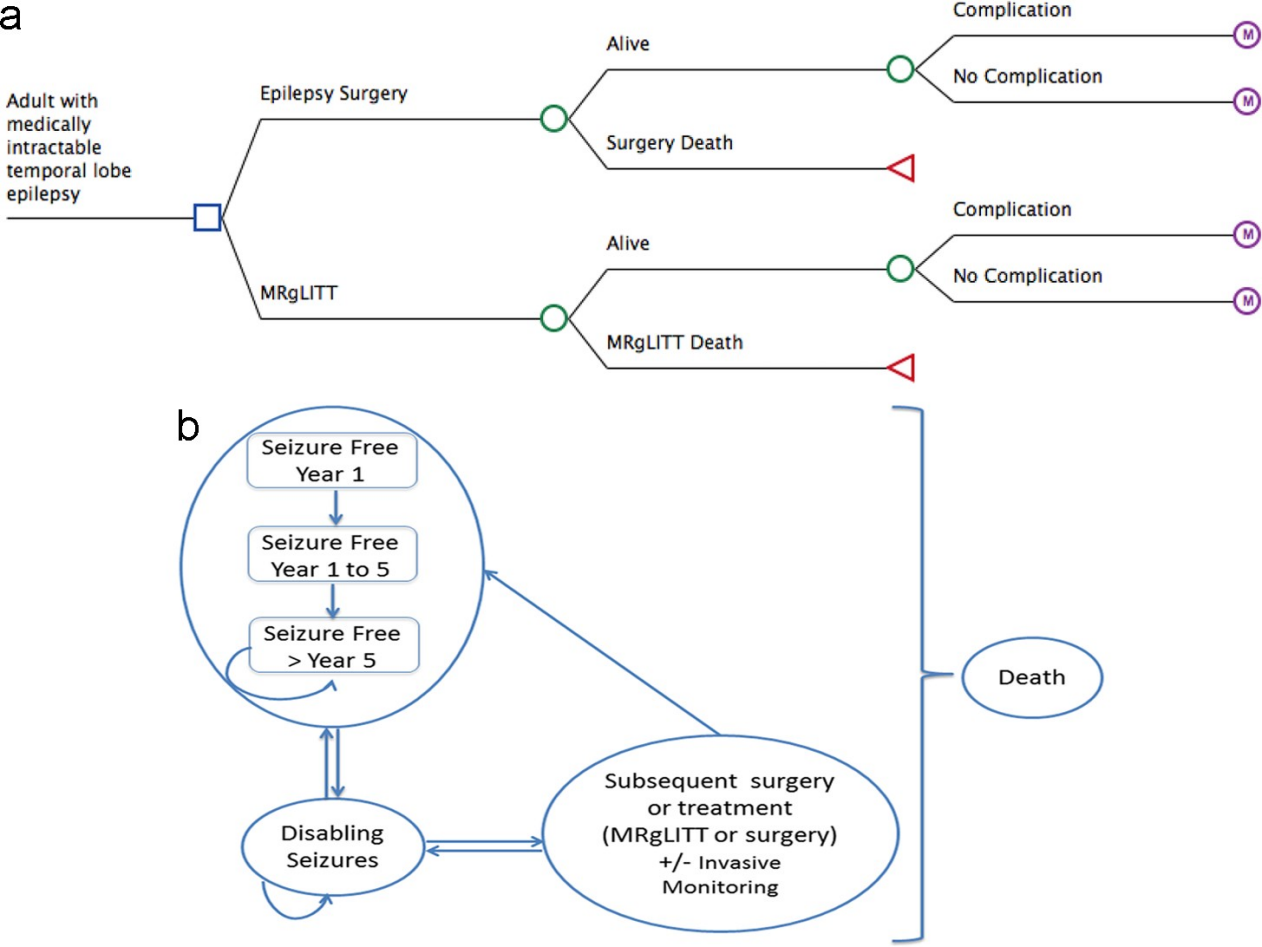
(a) Decision Tree and (b) health states for MRI-guided laser interstitial thermal therapy (MRgLITT) versus epilepsy surgery.

A patient who survived MRgLITT or surgery could develop complications from the treatment (Fig 1). During each yearly cycle, a patient could move among a pre-specified set of health states: seizure-free (defined as entirely seizure-free or have auras only), or disabling seizures. From the seizure-free health state, the patient could transition to disabling seizures state, or remained seizure-free. Following an unsuccessful surgery and the patient experienced disabling seizures, the patient could undergo subsequent surgery (with or without invasive monitoring) or continue with AEDs. The number of subsequent surgery was limited to a maximum of two, occurring within 10 years from the initial surgery [13–16]. Following an unsuccessful MRgLITT, a patient could undergo up to two subsequent treatments (either MRgLITT or surgery, with a maximum of one surgery) within 5 years from the initial MRgLITT [9, 10, 17, 18]. A patient who underwent a subsequent procedure and survived could develop a complication from the subsequent procedure, and may become seizure-free or continue to have disabling seizures, and transition between these two health states. The patient was followed until death occurred, either from epilepsy-related mortality or other causes of death.

The model did not consider non-resective palliative surgery such as vagal nerve stimulation or deep brain stimulation. MRgLITT and surgery could result in death or complications related to the procedure. The model considered permanent neurological complications after surgery or MRgLITT, such as visual field deficit, by incorporating utilities associated with permanent neurological complications, as these could exert a long-term impact on patients’ quality of life [19]. Utilities associated with transient complications (e.g., infection) were not considered, as these transient events limit their impact on life-time health outcomes (none have been reported to result in death) [19].

The model was constructed and analyzed using TreeAge Pro (TreeAge software, Williamstown, Mass).

### Model inputs

We conducted a MEDLINE literature search for systematic reviews to identify data to populate our model. When systematic reviews were not available, we searched for individual studies on surgery and MRgLITT (S1 Fig). Data from individual studies were synthesized through a meta-analysis with random effects model due to study heterogeneity, using Comprehensive Meta-Analysis (CMA, Englewood).

#### Probability of seizure-control

The probability of being seizure-free at 1-year following surgery [0.652 (95% CI: 0.567–0.728)] was based on the study by Jain *et al*. [20]. We used the study by Choi *et al*. [21] for the probabilities of (i) becoming seizure-free after experiencing disabling seizures annually the first 5 years after surgery [0.059 (95% CI: 0.009–0.110)] and more than 5 years after surgery [0.020 (95% CI: 0.002–0.072)], and (ii) returning to disabling seizures from seizure-free state the first 5 years after surgery [0.056 (95% CI: 0.029–0.083)] and more than 5 years after surgery [0.042 (95% CI: 0.016–0.068)] (Table 1). The probability of being seizure-free at 1-year after MRgLITT was based on Wu *et al*. [22] and Kang *et al*. [23] [0.580 (95% CI: 0.400–0.600)]. Since no literature data were available on the probabilities of becoming seizure-free from disabling seizures state, and returning to disabling seizures from seizure-free state post MRgLITT, we assumed that these probabilities were similar to surgery. The probability of being seizure-free after subsequent surgery was obtained from Krucoff *et al*. [24] [0.470 (95% CI: 0.376–0.564)]. Due to paucity of data on the probability of being seizure-free after a subsequent MRgLITT, we assumed that the probability was similar to subsequent surgery.

**Table 1:** Probability parameters for model.

Parameter	Subparameter	Base case value; SD (95% CI)	Probability Distribution	Data Source
Probability of Surgical Complication		0.030; 0.01 (0.020, 0.050)	Beta	Hader et al. 2013[25]
Probability of Subsequent Surgical Complication		0.030; 0.01 (0.020, 0.050)	Beta	Hader et al. 2013[25]
Probability of Surgery complication after MRgLITT		0.030; 0.01 (0.020, 0.050)	Beta	Hader et al. 2013[25]
Probability of MRgLITT Complications		0.030; 0.01 (0.020, 0.050)	Beta	Hader et al. 2013[25]
Probability of Subsequent MRgLITT Complication^§^		0.030; 0.01 (0.020, 0.050)	Beta	Hader et al. 2013[25]
Probability of Subsequent Surgery		0.050; 0.026 (0.039, 0.084)	Beta	Germano et al 1994, Goellner et al. 1994, Jung et al. 2013, Salanova et al. 2005[13–16]
Probability of Subsequent Treatment Post MRgLITT		0.196; 0.094 (0.045, 0.473)	Beta	Willie et al. 2014, Kang et al. 2016, Gross et al. 2018, Tao et al. 2018[9, 10, 17, 18]
Probability of Subsequent MRgLITT		0.462; 0.162 (0.139, 0.820)	Beta	Willie et al. 2014, Kang et al. 2016, Tao et al. 2018[9, 10, 18]
Probability of invasive monitoring with Subsequent Surgery/ MRgLITT		0.164; 0.009 (0.148, 0.182)	Beta	Krucoff et al. 2017[24]
Probability of death after initial and subsequent surgery		0.003; 0.002 (0, 0.0075)	Beta	Choi et al. 2008[21]
Probability of death after initial and subsequent MRgLITT^§§^		0.000; 0.0002 (0, 0.0075)	Beta	Wu et al. 2019[22]
Probability of SF in year 1:	Surgery	0.652; 0.038 (0.567, 0.728)	Beta	Jain et al. 2018[20]
	MRgLITT	0.580; 0.030 (0.400, 0.600)		Wu et al. 2019, Kang et al. 2017[22, 23]
Probability of SF with subsequent surgery or MRgLITT in year 1	Surgery	0.470; 0.047 (0.376, 0.564)	Beta	Krucoff et al. 2017[24]
	MRgLITT	0.470; 0.047 (0.376, 0.564)	Beta	Krucoff et al. 2017[24]
Transition probability for Surgery and MRgLITT^¶^ from:	SF to DS year 1–5	0.056; 0.014 (0.029, 0.083)	Beta	Choi et al. 2008[21]
	SF to DS after year 5	0.042; 0.013 (0.016, 0.068)	Beta	Choi et al. 2008[21]
	DS to SF year 1–5	0.059; 0.026 (0.009, 0.110)	Beta	Choi et al. 2008[21]
	DS to SF after year 5	0.020; 0.026 (0.002, 0.072)	Beta	Choi et al. 2008[21]
Standardized mortality ratios:	Seizure Free	1.11; 0.778 (0.63, 1.93)	Normal	Choi et al. 2008[21]
	DS after Surgery	5.42; 1.293 (3.97, 7.77)	Normal	Choi et al. 2008[21]
	DS after Laser^¶¶^	5.42; 1.293 (3.97, 7.77)	Normal	Choi et al. 2008[21]

SF = seizure-free; DS = disabling seizures; MRgLITT = MRI-guided laser interstitial thermal therapy

^**§**^Assumed probability of subsequent MRGLITT complication was the same as initial MRIGLITT complication.

^§§^Probability of death from MRgLITT assumed to be the same as surgery

^**¶**^Transition probability between SF and DS for MRgLITT assumed to be the same as surgery

^**¶¶**^Standardized mortality ratios for disabling seizures following MRgLITT assumed to be the same as surgery

SD = standard deviation

#### Probabilities of initial surgery or MRgLITT complications and death

We used data from a systematic review/meta-analysis for the probability of experiencing a neurological complication from the first surgery [0.030 (95% CI: 0.020–0.050)] [25]. There was variability in the reported neurological complications following MRgLITT, and frequently there was lack of clarity as to whether the complication was transient or permanent. We assumed that the probability of a neurological complication after MRgLITT was similar to surgery.

The probability of death following surgery was derived from Choi *et al*. [21] [0.003 (95% CI: 0–0.008)]. MRgLITT is a relatively new procedure and no death has been reported [22].

#### Probabilities of subsequent surgery or MRGLITT, complications and death

We estimated the probability of subsequent surgery was 0.050 (95% CI: 0.039–0.084) [13–16], and the probability of subsequent treatment (either MRgLITT or surgery) following an initial unsuccessful MRgLITT was 0.196 (95% CI: 0.045–0.473) [9, 10, 17, 18]. The probability of complications and death following subsequent surgery and subsequent MRgLITT were assumed the same as the initial surgery and initial MRgLITT, due to lack of published data.

#### Mortality for seizure-free and disabling seizures states

Patients with drug resistant epilepsy have a higher mortality rate compared to the general population [26]. We used estimates from Choi *et al*. [21] who pooled data to derive standardized mortality rates for seizure-free and disabling seizures states following surgery, and applied the same estimates for MRgLITT. The probability of all-cause mortality was obtained from Statistics Canada age-specific mortality rates [27].

#### Costs

We used data from the Ontario Case Costing Initiative (OCCI), an in-patient patient-level costs database from the Ontario Ministry of Health and Long-Term Care, to estimate hospital costs associated with surgery (Table 2) [28]. Physician costs, including surgeon, assistant, and anesthesia, associated with surgery were obtained from the Ontario Schedule of Benefits [29].

**Table 2:** Cost parameters for model.

Parameter	Subparameter	Mean Value; SD (Upper and Lower Range) ^§^	Probability Distribution	Data Source
***MRgLITT Costs*:** MRgLITT system* ^a^	Capital costs	$434,000	Gamma	Communication with Monteris Medical
	Annual service plan	$62,000	Gamma	Communication with Monteris Medical
	Disposable equipment (per patient)	$15,624	Gamma	Communication with Monteris Medical
	CT head with contrast	$500	Gamma	New Choice Health[30]
	MRI head pre- and post- procedure	$1500/case	Gamma	New Choice Health[30]
	MRgLITT hospitalization^†^	$1,939	Gamma	OCCI[28]
***MRgLITT Costs*:** Physician costs for MRgLITT	Surgeon^††^	$1551.20	Gamma	SOB[29]
	Anaesthesia^††^	$330.22	Gamma	SOB[29]
	Assistant^††^	$204.68	Gamma	SOB[29]
	CT head complex with IV contrast (X405)	$75.85	Gamma	SOB[29]
	MRI preprocedure (X421)	$73.35	Gamma	SOB[29]
	MRI post-procedure (X425)	$36.70	Gamma	SOB[29]
***MRgLITT Costs*:** Total (Disposable equipment, hospitalization, imaging, physician) ^§§^		$21,835; $1,553 ($10,918, $32,753)	Gamma	
Cost of MRgLITT Complication^‖^ ^b^*^§§^		$2,200; $550 ($1,100, $3,299)	Gamma	OCCI[28]
***Pre-Surgical Evaluation before Subsequent Surgery/ MRgLITT Costs***	Hospitalization	$10,998	Gamma	OCCI[28]
	Prolonged EEG:** Technical	$597	Gamma	SOB[29]
	Prolonged EEG:** Professional	$970	Gamma	SOB[29]
	MRI: Technical	$750	Gamma	New Choice Health[30]
	MRI: Professional (X421)	$73.35	Gamma	SOB[29]
	Total^§§^	$13,389; $3,347 ($6,694, $20,083)	Gamma	
***Surgery Costs*:** Surgery hospitalization		$17,561	Gamma	OCCI[28]
***Surgery Costs*:** Physician costs	Surgeon	$2184.20^††^	Gamma	SOB[29]
	Anaesthetist	$435.29	Gamma	SOB[29]
	Assistant	$385.28	Gamma	SOB[29]
	Total cost of surgery^**§§**^	$20,566; $5,141 ($10,283, $30,849)	Gamma	SOB[29]
	Cost of surgical complication ^b^*^§§^	$17,561; $4,390 ($8,781-$26,342)	Gamma	OCCI[28]
***Invasive Monitoring Costs*:** Hospitalization		$25,760	Gamma	OCCI[28]
***Invasive Monitoring Costs*:** Physician costs	Surgeon	$1,168	Gamma	SOB[29]
	Anaesthetist	$210	Gamma	SOB[29]
	Assistant	$157	Gamma	SOB[29]
	Total^**§§**^	$27,295; $6,824 ($13,647, $40,942)	Gamma	
***Health Resource Use (HRU***: Surgery and MRGLITT ^‖^ *^c^	Seizure-free year 1^**§§**^	$6,256; $1,564 ($3,128, $9,384)	Gamma	OCCI, SOB, Langfitt et al. 2007, ODB[28, 29, 31, 32]
	Seizure-free year 2^**§§**^	$3,554; $889 ($1,777, $5,331)	Gamma	OCCI, SOB, Langfitt et al. 2007, ODB[28, 29, 31, 32]
	Seizure-free after year 2^**§§**^	$2,804; $701 ($1,401, $4,205)	Gamma	OCCI, SOB, Langfitt et al. 2007, ODB[28, 29, 31, 32]
	Disabling Seizure (DS)^**§§**^	$6,377; $1,594 ($3,188, $9,565)	Gamma	OCCI, SOB, Langfitt et al. 2007, ODB[28, 29, 31, 32]

^§^Upper and lower range of costs based on 50% above and below the mean cost

^§§^Costs included in the probabilistic sensitivity analysis

*converted to Canadian dollars using the Bank of Canada currency converter (accessed February 3, 2018)

^†^based on OCCI data on cost of hospitalization for epilepsy surgery for 1 day, excluding intensive care unit stay

^††^based on Schedule of Benefits code N124—Functional stereotaxy

^‡^based on Schedule of Benefit code- X481 MRI guidance of biopsy or lesion ablation, internal organ

^‖^MRgLITT cost assumed to be same as for epilepsy surgery

^a^ 5% federal purchasing tax applied; linear depreciation of capital cost of MRgLITT equipment over 10 years, with discount rate of 1.5%

^b^ Difference in atypical (13 days) vs. typical (6.5 days) length of hospitalization is 6.5 days

**Based on 12units/day and 5.5 day length of stay from OCCI

^c^Number of units for each category of health resource use (e.g. 2 inpatient events) was derived from study by Langfit et al. 2007, multiplied by cost/unit, which was obtained from Ontario Case Costing Initiative (OCCI) or Schedule of Benefit (SOB) or Ontario Drug Benefit (ODB) Formulary

SD = standard deviation

We obtained capital cost of MRgLITT equipment (Can $434,000) and yearly equipment service plan cost (Can $62,000) from the manufacturer, Monteris Medical (personal communication in November 2018). The capital cost of MRgLITT equipment was depreciated linearly over 10 years to account for the decline in capital asset of the MRgLITT equipment as the equipment has a limited useful lifespan. Both capital and annual equipment service plan costs were distributed amongst the simulated patients. A 5% Canadian federal tax was applied for the purchase of hospital medical devices and services. Capital, equipment service plan, and disposable equipment costs, were converted to Canadian dollars from US dollars. For physician reimbursement, we used fees for similar procedures from the Schedule of Benefits [29]. Technical fees for head CT and MRI were estimated from the average costs of these imaging modalities in Ontario [30]. Distributions of the costs of MRgLITT were included as costs may vary across manufacturers, and we anticipate that the capital costs of equipment, yearly equipment service plan, and disposable equipment costs may change as more centers utilize MRgLITT for the treatment of TLE.

Patients undergoing MRgLITT generally are hospitalized for 24 hours (with no neurosurgical intensive care unit [ICU] stay). Hospitalization cost of MRgLITT was estimated from the difference between total hospitalization cost for epilepsy surgery and neurosurgery ICU cost, and averaging this cost over the total length of stay for surgery hospitalization [28].

Costs of surgery complications were assumed to increase the length of stay twice more than the typical epilepsy surgery hospitalizations (13 days vs. 6.5 days respectively) [28]. Costs of MRgLITT complications were estimated on the assumption that one additional day of hospitalization was required as a result of the complication.

For the quantity of healthcare resources used, including outpatient visits, hospitalizations, emergency department visits, diagnostic tests, and AEDs, at one year, two year and more than two years after surgery, we used data from Langfitt and colleagues (31) (S1 Table). Costs per unit of healthcare resources used were derived from the OCCI [28], Schedule of Benefits [29], and Ontario Drug Benefit formulary [32]. We assumed that healthcare resource use following MRgLITT was similar to surgery.

#### Utilities

Utility estimates for the seizure-free and disabling seizures states for surgery, with complications [0.77 (95% CI: 0.32–1.0) and 0.66 (95% CI: 0.19–1.0) respectively] and without complications [0.97 (95% CI: 0.87–1.0) and 0.78 (95% CI: 0.41–1.0) respectively], were obtained from Choi *et al*. [21] Using standard gamble, the authors interviewed patients who had undergone TLE surgery to elicit their preferences for different health states [21]. The utility estimates following MRgLITT were assumed the same as surgery, as the impact of seizure outcomes and possible complications were similar (Table 3).

**Table 3:** Utility parameters for model.

Parameter	Subparameter	Mean value; SD (95% CI)	Probability Distribution	Data Source
Utilities of seizure-free Surgery and Subsequent Surgery	No surgical complication	0.97; 0.02 (0.87, 1.0)	Beta	Choi et al. 2008[21]
	Surgical complication	0.77; 0.12 (0.32, 1.0)	Beta	Choi et al. 2008[21]
Utilities of seizure-free MRgLITT and Subsequent MRgLITT*	No MRgLITT complication	0.97; 0.02 (0.87, 1.0)	Beta	Choi et al. 2008[21]
	MRgLITT complication	0.77; 0.12 (0.32, 1.0)	Beta	Choi et al. 2008[21]
Utilities of disabling seizures Surgery and Subsequent Surgery	No surgical complication	0.78; 0.11 (0.41, 1.0)	Beta	Choi et al. 2008[21]
	Surgical complication	0.66; 0.17 (0.19, 1.0)	Beta	Choi et al. 2008[21]
Utilities of disabling seizures MRgLITT and Subsequent MRgLITT*	No MRgLITT complication	0.78; 0.11 (0.41, 1.0)	Beta	Choi et al. 2008[21]
	MRgLITT complication	0.66; 0.17 (0.19, 1.0)	Beta	Choi et al. 2008[21]

* assumed to be same as surgery

SD = standard deviation

### Analyses

#### Base-case analysis

We conducted a microsimulation to evaluate the expected QALYs, incremental QALYs, costs, incremental costs, and ICER, of a hypothetical cohort of adults with drug resistant TLE, using 50,000 individual patient iterations.

#### Sensitivity analyses

We executed a series of one-way, threshold, and probabilistic sensitivity analyses to examine the impact of uncertainty in model parameters. One-way sensitivity analyses were performed for each parameter over plausible ranges, and presented as a Tornado diagram. Threshold analyses were conducted to identify influential parameters for the model, i.e. those with thresholds within plausible ranges. The value of each parameter was varied over a broad range to determine if the preferred strategy changed, and if it did, the threshold value for that parameter was determined. A cost-effectiveness threshold (CET) of $50,000/QALY was assumed to calculate the net monetary benefit (NMB). NMB assesses the difference between QALY for a given CET threshold ($50,000/QALY), and cost (NMB = QALYxCET–incremental cost) for MRgLITT relative to surgery, to scale the benefits in monetary units.

Probabilistic sensitivity analysis (PSA) was performed to assess parameter uncertainty, by simultaneously varying the parameters according to pre-specified distributions, using 7,000 samples and 1000 iterations. The following distributions were assigned for parameter estimates: i) beta distribution for probabilities and utilities; ii) log-normal distribution for standardized mortality rates; and iii) gamma distribution for costs. The results of the PSA were presented as a cost-effectiveness acceptability curve (CEAC) with a series of cost-effectiveness threshold (CET) from $0 to $120,000 per QALY.

#### Scenario analyses

Following an unsuccessful MRgLITT and the patient continued to have disabling seizures, the patient could undergo subsequent MRgLITT or surgery. To determine which subsequent treatment was optimal, we evaluated the costs and effects of different options of subsequent treatments, that is, up to two subsequent MRgLITT and no subsequent surgery, one subsequent surgery only, or no subsequent treatment.

#### Value of information (VOI)

We conducted a VOI analysis to evaluate the value of conducting additional research to eliminate uncertainty for a decision based on current, imperfect data [33]. The VOI is the difference in net-monetary-benefit (NMB) of the optimal strategy given perfect information versus the strategy that would be adopted given current information. First, we assessed the expected value of perfect information (EVPI) by averaging the maximum NMB over the joint distribution of all the parameters in the model. Next, we evaluated the expected value of partial perfect information (EVPPI) for the probabilities associated with MRgLITT. Since MRgLITT is a relatively new treatment, there is larger uncertainty relating to the probabilities associated with MRgLITT, including seizure-free outcome following the initial and subsequent MRgLITT, becoming seizure-free from disabling seizures state, returning to disabling seizures from seizure-free state, complications and death from MRgLITT, and subsequent treatment after a failed MRgLITT. We also evaluated the utilities associated with MRgLITT due to great uncertainties in the utilities of seizure-free and disabling seizures states associated with MRgLITT.

Since information could be of value to more than one individual, we assessed EVPI and EVPPI for the individual patient, as well as the Ontario population who stand to benefit from MRgLITT. The individual EVPI and EVPPI were assessed over a life-time horizon. For the population EVPI and EVPPI, we used a 5-year (from 2019 to 2024) and 10-year time-horizon (from 2019 to 2029), as we assumed that this was the period over which new information on this technology would be of interest to the healthcare payer. The population EVPI and EVPPI were calculated by multiplying the individual EVPI and EVPPI with the number of patients who were eligible for MRgLITT. The number of eligible patients were estimated from: prevalence (96.8/1,000) and incidence (0.45/1,000) rate of epilepsy [34] multiplied by the adult population of Ontario [35] (accounting for a population growth rate of 0.8%/year) [36], and the proportion of epilepsy patients who were drug resistant (30%) [1], and eligible for MRgLITT/surgery (40%) [37].

## Results

### Base-case analysis

The expected life-years following MRgLITT (26.43 life-years) was minimally lower than following surgery (26.44 life-years) (Table 4). The expected QALYs after MRgLITT (24.70 QALYs) were higher than surgery (24.62 QALYs). The cost of MRgLITT ($165,303) was also higher than surgery ($157,482). MRgLITT yielded 0.08 more QALYs, cost $7,821 more, and had ICER of $94,350/QALY compared to surgery.

**Table 4:** Base case analysis.

Treatment strategy	Life-years	Costs	Incremental costs	QALYs	Incremental QALYs	Incremental cost effectiveness ratio (ICER)	Incremental NMB
MRgLITT	26.43	$165,303	$7,821	24.70	0.08	$94,350/QALY	-$3,821
Surgery	26.44	$157,482		24.62			

### Sensitivity analyses

**Fig 2:**
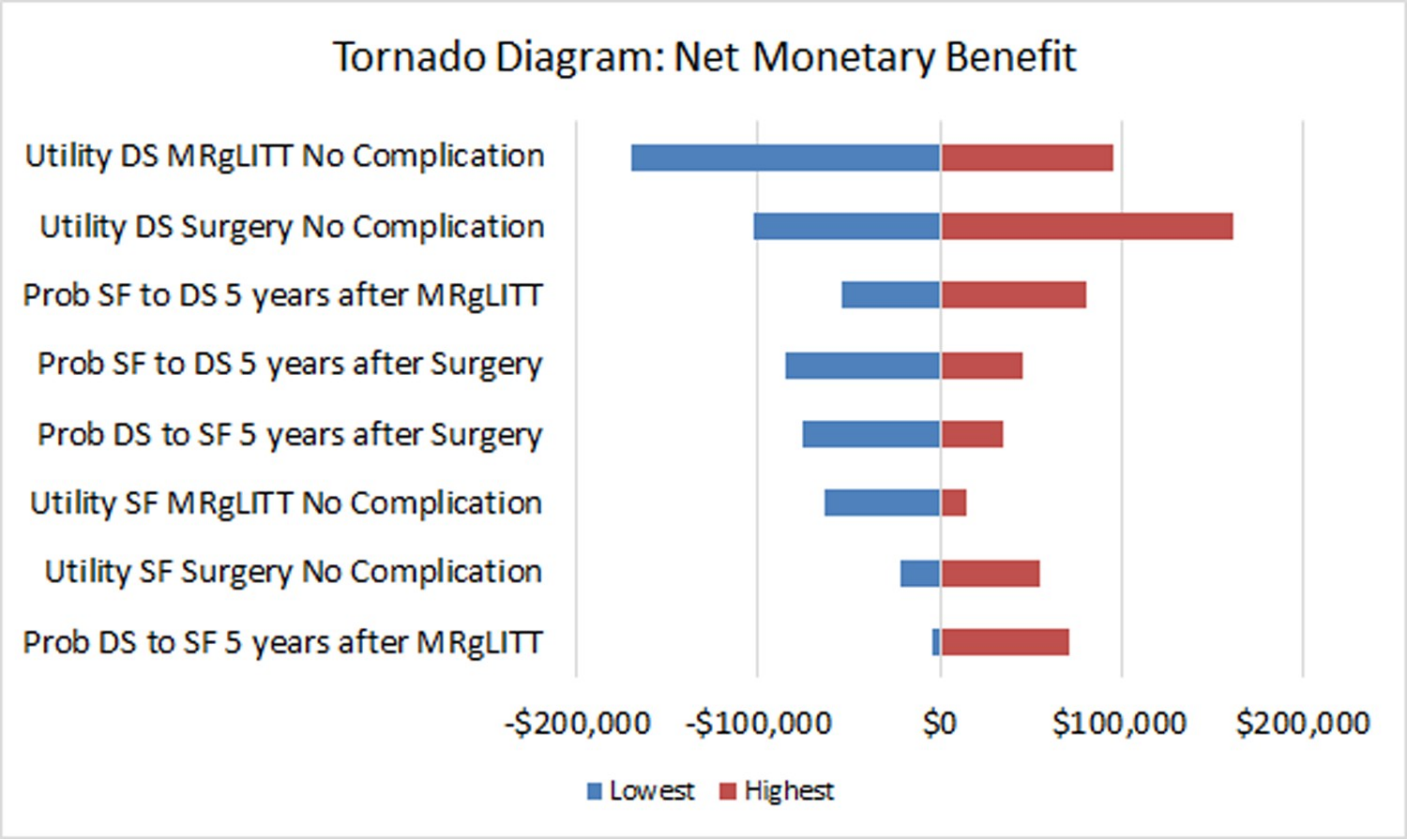
Tornado diagram from one-way sensitivity analyses showing the incremental net monetary benefit (NMB) of MRgLITT versus surgery. Influential parameters include utility of disabling seizures state after MRgLITT as well as surgery and without complication, probability of returning to disabling seizures from seizure-free state 5 years after surgery as well as MRgLITT, probability of becoming seizure-free from disabling seizures state 5 years after surgery as well as MRgLITT, and utility of seizure-free after surgery without complication. (SF: seizure-free; DS: disabling seizures).

One-way sensitivity analyses revealed that the most influential parameters were utilities of disabling seizures state after MRgLITT and surgery without complication, probabilities of returning to disabling seizures from seizure-free state 5 years after surgery and MRgLITT, probabilities of becoming seizure-free from disabling seizures state 5 years after surgery and MRgLITT, and utilities of seizure-free after surgery without complication (Fig 2).

Thresholds were identified for the following parameters: probability of becoming seizure-free from disabling seizures state 5 years after surgery and MRgLITT, probability of returning to disabling seizures from seizure-free state 5 years after surgery and MRgLITT; cost of MRgLITT disposable equipment, utilities of disabling seizures after surgery or MRgLITT, and seizure-free state after surgery or MRgLITT without complication (S2 Table).

**Fig 3:**
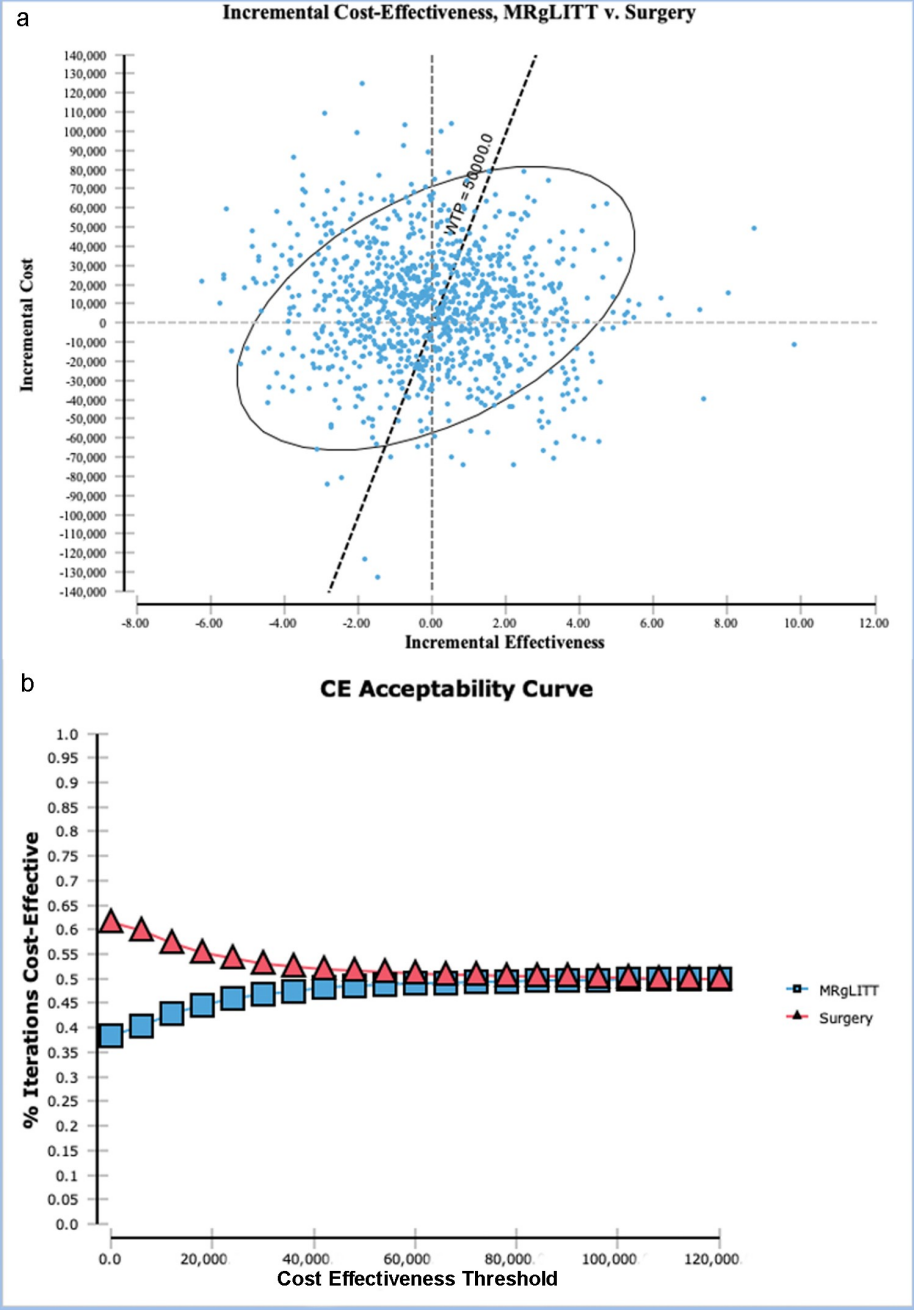
(a) Incremental cost-effectiveness ratio of MRgLITT vs. surgery scatter plot from probabilistic sensitivity analysis shows that 51.4% of the iterations are above the cost-effectiveness threshold (CET) of $50,000/QALY. (b) The cost-effectiveness acceptability curve (CEAC) demonstrates that for the range of cost-effectiveness threshold (CET) from $0 to $120,000/QALY, surgery is the preferred strategy in more than 50.0% of iterations.

PSA of MRgLITT versus surgery showed that 51.4% of the iterations were above the CET of $50,000/QALY (Fig 3A). The CEAC demonstrated that for the range of CET from $0–$100,000/QALY, surgery was the preferred strategy in more than 50.0% of iterations (Fig 3B).

### Scenario analyses

Following an unsuccessful MRgLITT, if the patient underwent up to two subsequent MRgLITT and no subsequent surgery, MRgLITT strategy resulted in lower costs and higher QALY than surgery strategy, with incremental NMB of $4,633 (Table 5). If the patient underwent one subsequent surgery only, the incremental NMB was higher relative to the two subsequent MRgLITT and no subsequent surgery option. If the patient did not undergo subsequent treatment following an unsuccessful MRgLITT, the MRgLITT strategy resulted in lower cost and lower QALY than surgery strategy, with lower incremental NMB of -$6,909.

**Table 5:** Scenario analyses of different subsequent treatment options following an unsuccessful MRgLITT.

Scenario	Treatment strategy	Costs	Incremental costs	QALYs	Incremental QALYs	Incremental NMB
Up to two subsequent MRgLITT	MRgLITT	$143,810	-$4,633	23.50	0.20	$4,633
	Surgery	$148,443		23.30		
One subsequent surgery only	MRgLITT	$141,110	-$7,311	23.34	0.02	$8,311
	Surgery	$148,421		23.32		
No subsequent treatment	MRgLITT	$131,489	-$17,091	22.85	-0.48	-$6,909
	Surgery	$148,580		23.33		

### Value of Information (VOI)

The expected monetary benefit of eliminating uncertainty for all parameters in the model (EVPI) varied from $8,277–$88,080 for the individual, $94.6–$1,006.5 million for the population for the 5-year time-horizon, and $116.8–$1,243.0 million for the population for the 10-year time-horizon, when the CET ranged from $0–$100,000/QALY (S2 Fig).

The value of additional research to eliminate uncertainty on probabilities associated with MRgLITT (EVPPI) varied from $576–$38,510 for the individual, and $6.6–$440.0 million for the population for the 5-year time-horizon, and $8.1–$543.5 million for the population for the 10-year time-horizon, when the CET ranged from $0–$100,000/QALY (S3 Fig).

The value of additional research to eliminate uncertainty on utilities associated with MRgLITT (EVPPI) varied from $0–$4,955 for the individual, and $0–$56.6 million for the population for the 5-year time-horizon, and $0–$69.9 million for the population for the 10-year time-horizon, when the CET ranged from $0–$100,000/QALY (S4 Fig).

## Discussion

Technological advances usually proceed at a much faster rate than the time it takes to rigorously evaluate the effectiveness of a new technology. MRgLITT is one such technological development, whereby the new treatment has been approved for clinical use by both U.S. Food and Drug Administration (FDA) and Health Canada, and has infiltrated clinical care, but there is no randomized controlled trial evaluating the efficacy of MRgLITT in drug resistant epilepsy. However, there is great enthusiasm to use this minimally invasive therapy as one of the treatment armamentaria for TLE. MRgLITT is increasingly used for the treatment of drug resistant TLE in the U.S. [9, 10, 17, 18, 38–41]. Few Canadian epilepsy centres have adopted this treatment as it is currently not reimbursed by the Ministry of Health and Long Term Care. Prior studies have compared the effectiveness, healthcare costs, or cost-effectiveness of epilepsy surgery to medical therapy in adults with drug resistant epilepsy, and demonstrated that epilepsy surgery was associated with higher QALYs and lower cost in the longer-term, particularly in those who were seizure-free [21, 31, 42, 43]. As yet, there is no published data on cost-effectiveness of MRgLITT versus epilepsy surgery.

In this early economic evaluation of MRgLITT, we found that MRgLITT resulted in more QALYs relative to epilepsy surgery. Although MRgLITT achieved slightly lower seizure freedom compared to surgery at 1-year follow-up, the higher probability of subsequent treatment if the initial MRgLITT failed to achieve seizure freedom compared to probability of subsequent surgery following an initial failed surgery, could have contributed to more QALYs gained in the MRgLITT strategy. We also found that despite the cost saving associated with reduced hospitalization during MRgLITT, the overall cost associated with MRgLITT was greater than surgery. The high capital, equipment service and disposable equipment costs of MRgLITT, in conjunction with the greater probability of subsequent treatment in the MRgLITT strategy, could have accounted for the higher cost of MRgLITT strategy.

In this study, we have not included medical treatment as a comparator. Our model assumes that patients are eligible for surgery or MRgLITT after evaluation for epilepsy surgery candidacy. Patients who are treated by medical therapy are those who after undergoing a standard surgical evaluation are not eligible for epilepsy surgery or MRgLITT due to inability to lateralize or localize the epileptogenic zone. Most patients who are found to be eligible for epilepsy surgery or MRgLITT are offered surgery or MRgLITT, and undergo one of these two treatments rather than medical therapy. As there is a potential that patients treated by medical therapy may be inherently different to those treated by surgery or MRgLITT, we have only considered patients who were eligible for both surgery and MRgLITT in the model, to allow for direct comparison of these two interventions. In the model, we have assumed that the patients have undergone the same pre-surgical diagnostic evaluation, since the choice of diagnostic evaluation may affect the costs of subsequent treatment strategies [44].

Previous economic evaluation of epilepsy surgery did not consider subsequent surgery following an initial failed surgery, despite the fact that subsequent surgery has been documented in the literature [24]. In this study, we modelled subsequent surgery or subsequent treatment following an initial failed surgery or MRgLITT respectively. Existing literature suggest that subsequent treatment following an initial failed MRgLITT [9, 10, 17, 18] was more common than subsequent surgery after a failed TLE surgery [13–16], for the following reasons. First, the probability of achieving seizure-freedom following MRgLITT [22] was lower than surgery [3, 4]. Second, MRgLITT is minimally invasive, requiring only a burr-hole as opposed to craniotomy for epilepsy surgery, thereby resulting in less scarring of brain and surrounding tissues. It could potentially be technically less challenging to undertake a repeat procedure after MRgLITT compared to surgery. Further, patients may be more willing to undergo a repeat procedure that is minimally invasive. We have also considered different subsequent treatment options after an initial failed MRgLITT, such as up to two subsequent MRgLITT, one subsequent surgery only or no subsequent treatment. We found that one subsequent surgery yielded the highest NMB, followed by up to two subsequent MRgLITT, and the no subsequent treatment option yielded the lowest NMB. Therefore, the one subsequent surgery is the optimal subsequent treatment option.

CET at which an intervention is considered as cost-effective varies across countries. With an ICER of $90,350/QALY, MRgLITT would not be considered as cost-effective at CET of $50,000/QALY, but would be regarded as cost-effective at higher CET of $100,000/QALY. Whilst cost-effectiveness of a new treatment is an important consideration for reimbursement decisions and uptake of the treatment in clinical practice, this is not the only consideration. Patient preferences for the treatment are also important considerations. Given that MRgLITT is minimally invasive, this treatment may be more acceptable to patients and could therefore lead to increase uptake of MRgLITT to mitigate the burden of drug resistant epilepsy. However, it is beyond the scope of this study to evaluate patient preferences.

MRgLITT is a relatively new intervention and there are no randomized controlled trials comparing MRgLITT to epilepsy surgery nor medical therapy. The effectiveness and safety profile of MRgLITT was based on data from observational studies or assumptions based surgical literature. Interpretations of these observational studies are limited by small sample size (most studies with less than 60 patients, except for one multi-center study with 234 patients [22]), single center results, short and variable follow-up periods, and inconsistent reporting of complications. There is usually a learning curve associated with any new procedure including MRgLITT. Effectiveness and complications of MRgLITT may potentially change over time as experience and knowledge on patient selection and techniques improve. On the other hand, epilepsy surgery is a well-established intervention, and two randomized controlled trials have reported on outcomes of TLE surgery. As well, there are more observational studies reporting on the effectiveness and safety of epilepsy surgery, with larger sample size and longer follow-up duration available on epilepsy surgery compared to MRgLITT. Since there is limited data on probabilities related to MRgLITT. Data input pertaining to MRgLITT was derived from observational studies or assumptions based on surgical literature. However, we conducted sensitivity analyses to examine uncertainty in parameter inputs. Further, we conducted VOI analysis to assess the expected cost of uncertainty with currently available data on the decision regarding MRgLITT versus surgery. The value of additional research to eliminate uncertainty of current information was $88,080 per individual at CET of $100,000/QALY (determined using EVPI). This cost was substantially higher compared to the estimated EVPI for atrial fibrillation therapies ($8,542) [45], and bronchodilators for chronic obstructive lung disease (€1,985) [46], supporting the value in allocating research on drug resistant TLE interventions. The expected monetary benefit of eliminating uncertainty for probabilities associated with MRgLITT was higher than for utilities associated with MRgLITT. Whilst there is value in conducting more research to reduce uncertainty on the probabilities and utilities associated with MRgLITT, the EVPPI indicates that priority should be given to additional research focusing on improving the precision of the estimates on probabilities related to MRgLITT. The population EVPPI was a conservative estimate, as the analysis included only patients in Ontario, whereas the new knowledge derived from additional research could be far reaching, and affect patients in other jurisdictions.

A potential limitation of the study is that the capital equipment cost of MRgLITT, yearly equipment service plan and per patient MRgLITT disposable equipment costs were derived from one manufacturer. To account for potential variations in these costs across manufacturers, we conducted sensitivity analyses. We found that variations in the capital cost of MRgLITT and yearly service plan cost did not affect the model’s decision, and MRgLITT still achieved more QALYs and costs more than surgery. However, the model was sensitive to variations in the cost of MRgLITT disposable equipment. When the cost of MRgLITT disposable equipment was below Can $12,244, MRgLITT was the preferred strategy, with higher QALYs gained and lower cost compared to surgery. Since MRgLITT is a relatively new therapy, few centres are utilizing this treatment for the management of TLE. It is foreseeable that as more centers utilize this treatment, the costs associated with MRgLITT, including disposable equipment cost will decrease, potentially changing the cost-effectiveness profile of MRgLITT relative to surgery.

## Conclusions

This early economic evaluation suggests that MRgLITT resulted in more QALYs gained and higher costs compared to surgery in the base-case. However, the model was sensitive to variations in the cost of MRgLITT disposable equipment. Further, this study showed that there is value in undertaking additional research, particularly to reduce uncertainty in the evidence on the effectiveness of MRgLITT. The findings of this study adds to the literature by demonstrating the cost-effectiveness of MRgLITT relative to surgery based on existing evidence, the influential parameters for the model and the opportunity cost of a decision based on current evidence, and hence the value of additional research on MRgLITT. Additional research such as a randomized controlled trial comparing the effectiveness of MRgLITT relative to epilepsy surgery could inform an updated health economic evaluation in the future, and support reimbursement decisions and diffusion of MRgLITT. Future research will include assessing the expected value of sample information to compare the costs and value of different trial designs for MRgLITT, to identify the trial with the greatest net benefit, and inform the optimal sample size for a future trial on MRgLITT.

## Supporting information

S1 Table Healthcare resource utilization after surgery and MRI-guided laser interstitial thermal therapy (MRgLITT). (DOCX)

S2 Table Threshold analysis for influential parameters. (DOCX)

S1 Fig PRISMA flow diagram of search strategy for MRgLITT literature from MEDLINE. Keywords for search include laser therapy and temporal lobe epilepsy.
(TIF)

S2 Fig The value of information analysis showing the expected monetary benefit of eliminating uncertainty for all the parameters (Expected Value of Perfect Information [EVPI]) for the cost-effectiveness threshold (CET) ranging from $0 to $100,000 per QALY. (a) The EVPI for the individual over a life-time horizon is $8,277–$88,080. The EVPI for the Ontario population (b) over a 5-year time-horizon is $94.6–$1,006.5 million, and (c) over a 10-year time-horizon is $116.8–$1,243.0 million.
(TIF)

S3 Fig The expected value of partial perfect information (EVPPI) for the probabilities associated with MRgLITT for the cost-effectiveness threshold (CET) ranging from $0 to $100,000 per QALY. (a) The EVPPI for the individual over a life-time horizon is $576–$38,510. The EVPPI for the Ontario population (b) over a 5-year time-horizon is $6.6–$440.0 million, and (c) over a 10-year time-horizon is $8.1–$543.5 million.
(TIF)

S4 Fig The expected value of partial perfect information (EVPPI) for the utilities associated with MRgLITT for the cost-effectiveness threshold (CET) ranging from $0 to $100,000 per QALY. (a) The EVPPI for the individual over a life-time horizon is $0–$4,955. The EVPPI for the Ontario population (b) over a 5-year time-horizon is $0–$56.6 million, (c) over a 10-year time-horizon is $0–$69.9 million.
(TIF)
