# Supplementary material for: Early economic evaluation of MRI-guided laser interstitial thermal therapy (MRgLITT) and epilepsy surgery for mesial temporal lobe epilepsy
Source: PLoS One. 2019 Nov 20;14(11):e0224571. doi: 10.1371/journal.pone.0224571 (PMC6867628; doi:10.1371/journal.pone.0224571)
Supplement: S1 Table — (DOCX) [file pone.0224571.s001.docx]

**S1 Table. Healthcare resource utilization after surgery and MRI-guided laser interstitial thermal therapy (MRgLITT)**

|  |  | **Seizure-Free** | | | | | | **Disabling seizures** | |
| --- | --- | --- | --- | --- | --- | --- | --- | --- | --- |
|  |  | **1^st^ year** | | **2^nd^ year** | | **After 2years** | |  | |
|  | **Cost/unit** | **Units** | **Total cost** | **Units** | **Total costs** | **Units** | **Total costs** | **Units** | **Total costs** |
| Outpatient | $84.95 | 12.6 | $1,070 | 9.6 | $816 | 9.6 | $816 | 9.5 | $807 |
| Inpatient | $10,998 | 2 | $880 | 0 | $0 | 0 | $0 | 2.1 | $1,155 |
| Laboratory test | $31 | 4.8 | $128 | 3.6 | $79 | 3.6 | $79 | 4.6 | $111 |
| Outpatient tests/ procedure | $74.4 | 3.5 | $676 | 3.6 | $220 | 3.6 | $220 | 3 | $185 |
| Emergency Department Visit | $425 | 2.2 | $168 | 0 | $0 | 0 | $0 | 4.9 | $208 |
| AEDs | $1955* | 1.7 | $3324 | 1.3 | $2,440 | 0.9 | $1,689 | 2 | $3,910 |
| Total |  |  | $6,256 |  | $3,554 |  | $2,804 |  | $6,377 |

Number of units of healthcare resource use was derived from Langfitt et al. 2007.(30) Costs per unit of healthcare resources used were derived from OCCI,(27) Schedule of Benefits,(28) and Ontario Drug Benefit formulary.(31) We assumed that costs related to healthcare resource use following MRgLITT were similar to surgery.

Healthcare resource use 2 years after surgery/ MRgLITT was assumed to be the same as for 2^nd^ year, except for AEDs, which decreased by the same proportion as between 1^st^ and 2^nd^ year after surgery/ MRgLITT.

*Cost/ unit for one year supply of anti-epileptic drugs (AEDs), based on average cost of 10 commonly used AEDs for temporal lobe epilepsy.
