# Supplementary material for: Early economic evaluation of MRI-guided laser interstitial thermal therapy (MRgLITT) and epilepsy surgery for mesial temporal lobe epilepsy
Source: PLoS One. 2019 Nov 20;14(11):e0224571. doi: 10.1371/journal.pone.0224571 (PMC6867628; doi:10.1371/journal.pone.0224571)
Supplement: S2 Table — (DOCX) [file pone.0224571.s002.docx]

**S2 Table. Threshold analysis for influential parameters**

| **Parameter** | **Threshold** |
| --- | --- |
| Probability of becoming seizure-free from disabling seizures state 5 years after surgery | 0.018 |
| Probability of becoming seizure-free from disabling seizures state 5 years after MRgLITT | 0.022 |
| Probability of returning to disabling seizures from seizure-free state 5 years after surgery | 0.044 |
| Probability of returning to disabling seizures from seizure-free state 5 years after MRgLITT | 0.041 |
| Cost of MRgLITT disposable equipment | $12,244 |
| Utilities of disabling seizures state after surgery without complication | 0.771 |
| Utilities of disabling seizures state after MRgLITT without complication | 0.789 |
| Utilities of seizure-free state after surgery without complication | 0.963 |
| Utilities of seizure-free state after MRgLITT without complication | 0.977 |
