# Supplementary figures and images for: Early economic evaluation of MRI-guided laser interstitial thermal therapy (MRgLITT) and epilepsy surgery for mesial temporal lobe epilepsy
Source: PLoS One. 2019 Nov 20;14(11):e0224571. doi: 10.1371/journal.pone.0224571 (PMC6867628; doi:10.1371/journal.pone.0224571)

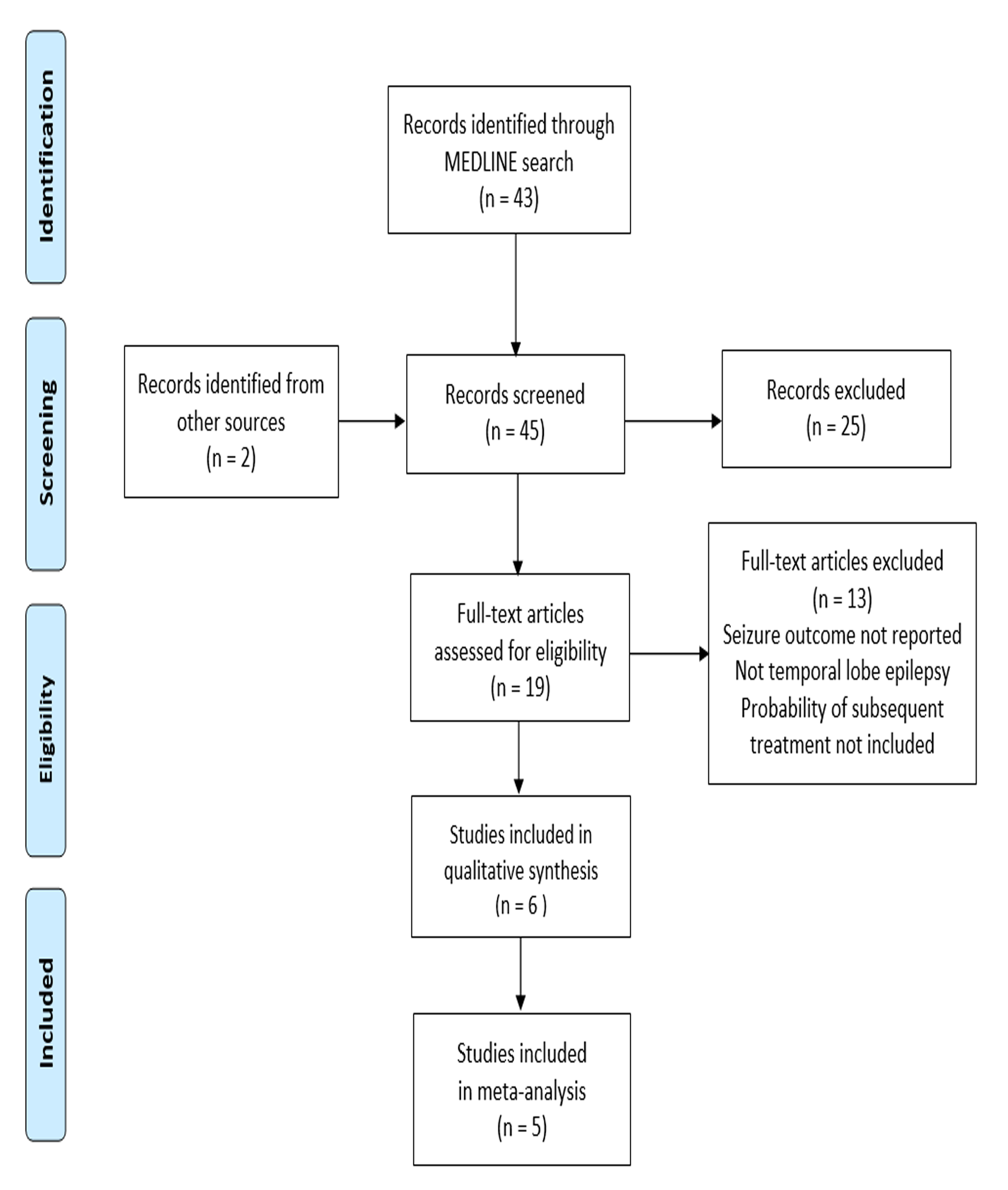

Supplement: S1 Fig — Keywords for search include laser therapy and temporal lobe epilepsy. (TIF) [file pone.0224571.s003.tif]

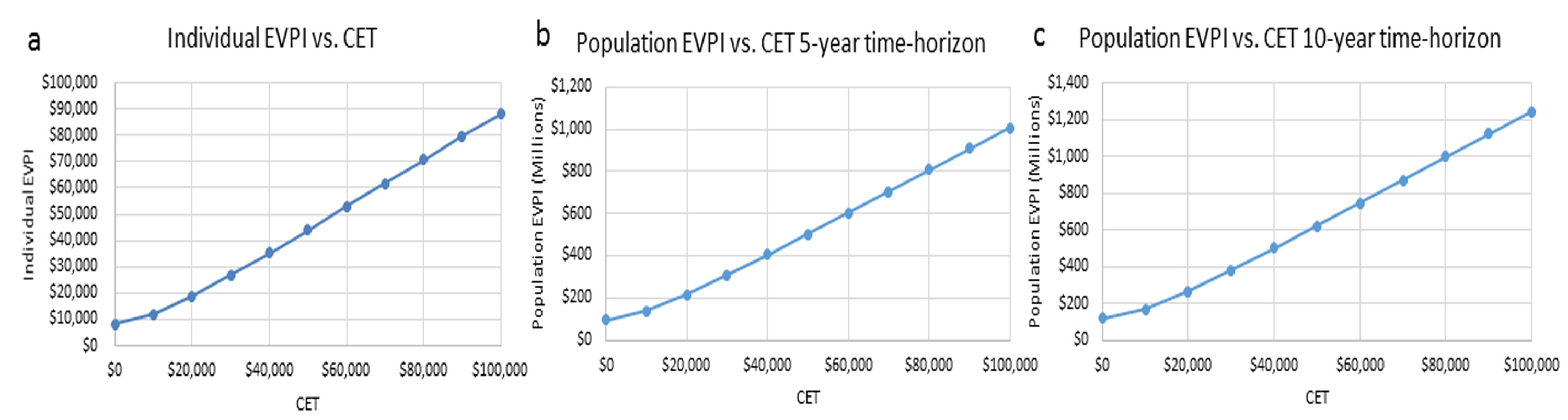

Supplement: S2 Fig — (a) The EVPI for the individual over a life-time horizon is $8,277–$88,080. The EVPI for the Ontario population (b) over a 5-year time-horizon is $94.6–$1,006.5 million, and (c) over a 10-year time-horizon is $116.8–$1,243.0 million. (TIF) [file pone.0224571.s004.tif]

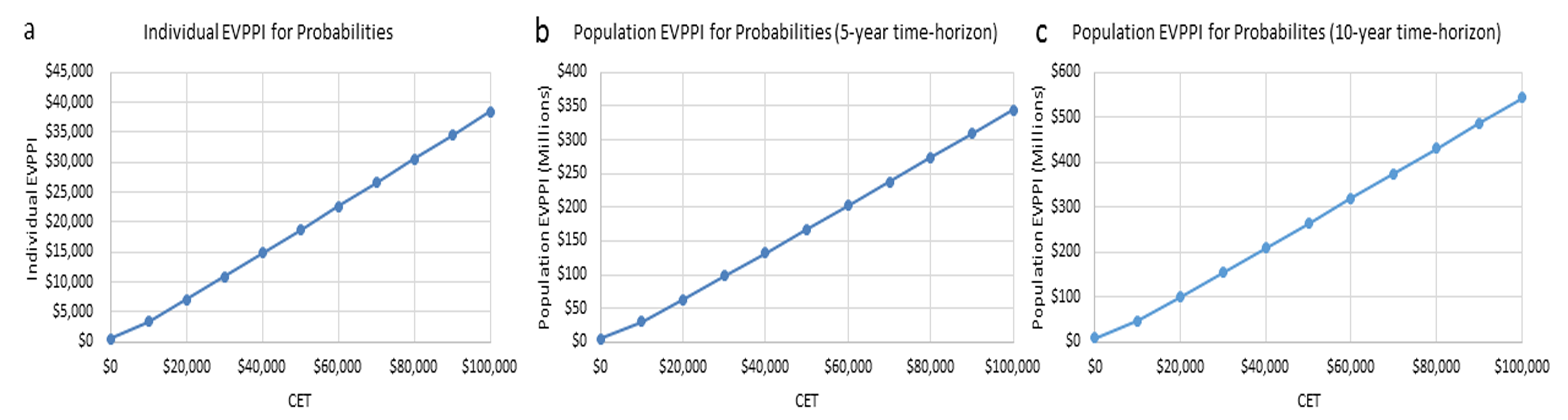

Supplement: S3 Fig — (a) The EVPPI for the individual over a life-time horizon is $576–$38,510. The EVPPI for the Ontario population (b) over a 5-year time-horizon is $6.6–$440.0 million, and (c) over a 10-year time-horizon is $8.1–$543.5 million. (TIF) [file pone.0224571.s005.tif]

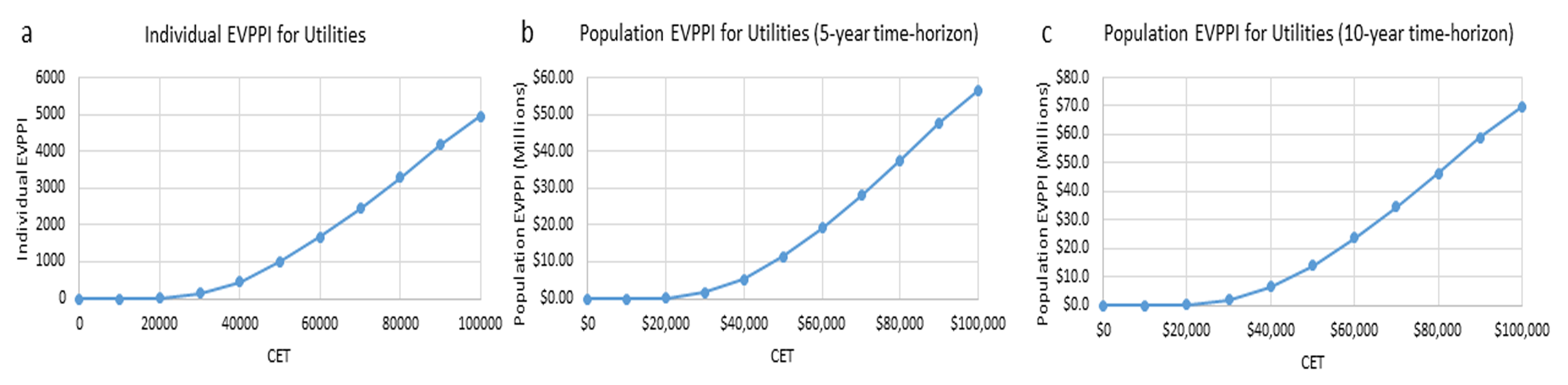

Supplement: S4 Fig — (a) The EVPPI for the individual over a life-time horizon is $0–$4,955. The EVPPI for the Ontario population (b) over a 5-year time-horizon is $0–$56.6 million, (c) over a 10-year time-horizon is $0–$69.9 million. (TIF) [file pone.0224571.s006.tif]
